# Dietary patterns and odds of Type 2 diabetes in Beirut, Lebanon: a case–control study

**DOI:** 10.1186/1743-7075-9-111

**Published:** 2012-12-27

**Authors:** Farah Naja, Nahla Hwalla, Leila Itani, Maya Salem, Sami T Azar, Maya Nabhani Zeidan, Lara Nasreddine

**Affiliations:** 1 Department of Nutrition and Food Sciences, Faculty of Agriculture and Food Sciences, American University of Beirut, P. O. Box 11–0236, Riad El-Solh, Beirut, 1107-2020, Lebanon; 2 Department of Internal Medicine, American University of Beirut, P.O.Box 11–0236, Riad El-Solh, Beirut, 1107-2020, Lebanon; 3 Members of the Public Health and Nutrition (PHAN) Research Group at the American University of Beirut, P.O.Box 11–0236, Riad El-Solh, Beirut, 1107-2020, Lebanon

**Keywords:** Dietary patterns, Lebanon, Type 2 diabetes

## Abstract

**Background:**

In Lebanon, Type 2 diabetes (T2D) has a major public health impact through high disease prevalence, significant downstream pathophysiologic effects, and enormous financial liabilities. Diet is an important environmental factor in the development and prevention of T2D. Dietary patterns may exert greater effects on health than individual foods, nutrients, or food groups. The objective of this study is to examine the association between dietary patterns and the odds of T2D among Lebanese adults.

**Methods:**

Fifty-eight recently diagnosed cases of T2D and 116 population-based age, sex, and place of residence matched control participants were interviewed. Data collection included a standard socio-demographic and lifestyle questionnaire. Dietary intake was evaluated by a semi-quantitative 97-item food frequency questionnaire. Anthropometric measurements including weight, height, waist circumference, and percent body fat were also obtained. Dietary patterns were identified by factor analysis. Multivariate logistic regression analysis was used to evaluate the associations of extracted patterns with T2D. Pearson correlations between these patterns and obesity markers, energy, and nutrient intakes were also examined.

**Results:**

Four dietary patterns were identified: Refined Grains & Desserts, Traditional Lebanese, Fast Food and Meat & Alcohol. While scores of the “Refined Grains & Desserts” had the highest correlations with energy (r = 0.74) and carbohydrates (r = 0.22), those of the “Fast Food” had the highest correlation with fat intake (r = 0.34). After adjustment for socio-demographic and lifestyle characteristics, scores of the Refined Grains & Desserts and Fast Food patterns were associated with higher odds of T2D (OR: 3.85, CI: 1.13-11.23 and OR: 2.80, CI: 1.14-5.59; respectively) and scores of the Traditional Lebanese pattern were inversely associated with the odds of T2D (OR: 0.46, CI: 0.22-0.97).

**Conclusions:**

The findings of this study demonstrate direct associations of the Refined Grains & Desserts and Fast Food patterns with T2D and an inverse association between the Traditional Lebanese pattern and the disease among Lebanese adults. These results may guide the development of nutrition interventions for the prevention and management of T2D among Lebanese adults.

## Introduction

Type 2 Diabetes (T2D) has reached epidemic proportions in both developed and developing countries and is now recognized as a major global health problem [1-4]. Lebanon, similar to other counties of the Middle East North Africa Region (MENA) region, is witnessing a polarization in disease patterns with under nutrition and its related diseases coexisting with chronic diseases such as T2D and cardiovascular diseases typically associated with developed countries [5]. Even though the prevalence of T2D among adults in Lebanon (15.8%) [6] appears to be lower than that reported from most other countries in the MENA region including Bahrain (25.5%), United Arab Emirates (23.3%) and Saudi Arabia (23.7%) [5], available data in the country suggested that prevalence rates of the disease are following an alarming trend over time, increasing from 11.6% in 1999 to 15.8% in 2004 [6,7]. According to the International Diabetes Federation, the projected prevalence of T2D among adults in Lebanon for year 2020 is 20.4% [8]. This is corroborated by a recent report suggesting that, worldwide, the greatest relative increase in the number of people with diabetes is expected to occur in countries of the Middle Eastern crescent [9]. This escalating trend in disease prevalence coupled to significant downstream pathophysiologic effects and enormous financial liabilities poses a major public health problem that necessitates the search for mitigating factors and strategies to address them.

Dietary intake is an important modifiable environmental risk factor in the development and prevention of T2D [10]. Although there is convincing evidence that excess consumption of calories and high glycemic index foods that are also low in phytochemical content are associated with an increased risk of T2D [11-14], the evidence regarding the association of other dietary related risk factors with T2D is far less complete [15]. More recently, several nutritional epidemiologic studies have examined the association between dietary patterns rather than single nutrients in relation to the risk of T2D. Dietary patterns may exert greater effects on health than individual foods, nutrients, or food groups [16]. This approach has been used to study obesity, diabetes, cancer, cardiovascular diseases, and all-cause mortality [17-24]. Most of the studies investigating the association of various dietary patterns with the risk of T2D –whether patterns were derived a posteriori (using cluster or factor analyses) or according to hypothesis-driven methods (such as index analysis) - showed that the adoption of a Western and Western-like dietary patterns, characterized by high intakes of processed foods, red meat, and refined grains, were related to a higher risk of glucose intolerance and T2D [17,21-27]. A recent study in Lebanon also showed that adherence to a Western dietary pattern was associated with higher odds of hyperglycemia and metabolic syndrome [28].

Furthermore, a Mediterranean dietary pattern, characterized by high consumption of fruits, vegetables, fish, and olive oil, has also been suggested to be protective against the risk of metabolic abnormalities and T2D [29-32]. In the East Mediterranean region, studies evaluating the association between the different dietary patterns and the risk of T2D are scarce [33,34].

The aim of this study was to examine the association of various dietary patterns with the odds of T2D among Lebanese adults. Findings from this study could inform prevention strategies among Lebanese adults with high risk of T2D.

## Materials and methods

This study was approved by the Institutional Review Board at the American University of Beirut. All participants signed a consent form prior to participation and had the right to withdraw from the study at any time.

### Study design

This is a case control study that investigated the association between dietary patterns and the odds of T2D among Lebanese adults. Eligible cases were patients older than 18 years of age, who were newly diagnosed with T2D (within 6 months of recruitment) and were attending the Private Clinics or the Dietary Department at the American University of Beirut Medical Center between April 2009 and December 2010. Controls were randomly selected from the general population and were matched to cases by age (± 5 years), sex, and area residence. Whenever a case was identified and recruited in the study, a control participant who is within ± 5 year-age interval of the case was recruited. For example, if a case was 45 years old, the matching control participant ought to be between 40 and 50 years of age. Matching for place of residence was performed, given that, in the Lebanese context, residents of the same of area tend to have comparable socioeconomic status [35]. After a case of T2D was identified in the clinical setting, a matching control was randomly chosen from the same or from the two adjacent residential buildings in which the case resided. In most buildings of Beirut, the names of residents are listed on an interphone list in the building entrance. The field worker will contact a random resident from this list and ask for the availability of a healthy adult who matches the case on age and sex. Should there be more than one adult in the household satisfying the inclusion criteria then only one is chosen. Controls had to be free of diagnosed diabetes. Two controls were selected to match each case. Recruitment of a matching control participant took place within two weeks of the case enrolment in the study. The sample size for this study (n = 180) was calculated using an alpha of 0.05, a power of 80%, and a standard effect (ω parameter described by Cohen 1988 [36]) of 0.5, detecting a minimum significant outcome measure (OR) of 2.44.

### Data collection

In a one to one interview, study participants completed a brief socio-demographic and lifestyle questionnaire and a semi quantitative Food Frequency Questionnaire (FFQ). In addition anthropometric measurements, including weight, height, waist circumference and percent body fat, were obtained. All the interviews were conducted by trained dieticians.

The sociodemographic and lifestyle questionnaire retrieved information regarding the following characteristics: age (continuous in years), sex, income per month (≤1.5 million L.L., >1.5 million L.L.; 1500 L.L is equivalent to almost $1 U.S.), marital status (single including single, divorced, and widowed, and married including currently married or living with a partner), education (less than high school education and greater than high school education), smoking (smokers including current smokers, non smokers including non-smokers and past smokers), physical activity (Low physical activity including light activities such as housework and casual walking and moderate to high physical activity including all other types of exercise such as biking, dancing, running, and aerobics), family history of T2D (a positive family history of T2D was indicated if the mother, father or both were diagnosed with the disease), frequency of breakfast consumption per week (continuous), and number of meals per day (continuous).

### Anthropometric measurements

Anthropometric measurements were obtained using standardized techniques and calibrated equipments. Participants were weighed to the nearest 0.1 kg in light indoor clothing and with bare feet or stockings. Using a stadiometer, height was measured without shoes and recorded to the nearest 0.5 cm. Body mass index (BMI) was calculated as the ratio of weight (kilograms) to square of height (meters squared). Waist circumference was measured using a plastic measuring tape, to the nearest 0.5 cm, at the midpoint between the bottom of the rib cage and above the top of the iliac crest during minimal respiration. Body fat was measured by the leg to leg bioelectric impedance (BIA) system (Tanita Body Fat Analyzer, TBF 105, Tanita Corporation of America, Inc., Arlington Heights, IL). Prior to BIA testing, participants were required to adhere to standard BIA testing guidelines, and bioelectrical impedance was measured in participants standing erect with bare feet on the analyzer's footpads. Fat Free Mass and percent body fat from BIA were calculated using the prediction equation supplied by the manufacturer. The validity of this method of body fat assessment was tested against underwater weighing and was shown to accurately assess body fat in men and women [37,38].

### Dietary intake assessment

Dietary intake of participants was assessed by a 97-item semi quantitative FFQ adapted from an instrument used in the total diet study of adult urban population in Beirut, Lebanon [39]. Further details about the development of the original questionnaire were described elsewhere [39]. The FFQ referred to food intake one year prior to diagnosis of T2D for cases and during the previous 12 months for controls. The questionnaire assessed the intake of commonly consumed food items grouped into 23 categories namely bread and cereals, grains and pasta, milk and dairy, fruits, juices, vegetables, potatoes, legumes, meat, poultry, fish, organ meat, eggs, fats and oils, caffeinated beverages, sweets and desserts, ice-cream, puddings, sugar derivatives, beverages, mixed dishes, fast food, and alcohol. Food items under each category were chosen to provide comprehensive representation of the Lebanese diet. Where possible, foods were separated into low fat and whole fat groups. The reference portions of the 2 dimensional food portion visual (Millen and Morgan, Nutrition Consulting Enterprises, Framingham, Massachusetts, United States) were used. Participants were asked to recall how often, on average, they consumed the specified portion size of every food item per day, per week, per month or never. Portion sizes, consumed from each food item, were then converted to daily portion intake. Daily energy and nutrient intakes were computed by multiplying the daily frequency of intake by the nutrient content of the specified portion size; using the food composition tables provided by N4 software (provided by N-squared Computing Nutritionist IV. Silverton (Oregon): N-squared Computing 1993) and using the food composition table of Middle Eastern foods for local and traditional dishes [40].

### Dietary patterns derivation

For the purpose of determination of dietary patterns, food items were grouped into twenty five food groups based on nutrient profile and culinary usage (Additional file 1: Appendix A). We have opted to include alcohol intake as a food group in the factor analysis rather than as a covariate in the association between patterns and T2D to allow for comparability with our previous studies assessing dietary patterns and diseases in Lebanon, where alcohol intake was also part of the factor analysis in dietary patterns derivation [28,41]. The total consumption for each food group was determined by summing the daily intake of servings from each item in this group. Food groups’ intakes were not adjusted for energy. Prior to running the factor analysis procedure, the correlation matrix between the twenty five food groups was visually and statistically examined to justify undertaking factor analysis. The chi square for Bartlett test of sphericity was significant at a p-value less than 0.05 and Kaiser-Meyer-Olkin test was greater than 0.6 [42]. The exploratory principal component factor analysis (PCFA) was implemented to identify dietary patterns using intake of the twenty five food groups. The number of factors retained was based on three criteria 1) the Kaiser criterion (eigenvalues >1), 2) inflection point of the scree plot, and 3) the interpretability of factors [43,44]. The derived dietary patterns were labeled based on food groups having a rotated factor loading greater than 0.4. Factor scores were calculated by multiple regression approach whereby independent variables in the regression equation are the standardized observed values of the items in the estimated patterns. These predictor variables are weighted by regression coefficients, which are obtained by multiplying the inverse of the observed variable correlation matrix by the matrix of factor loadings. The factor scores are the dependent variables in the regression equation [45]. Each individual received a factor score for each dietary pattern. Further details about the derivation of dietary patterns are described elsewhere [28].

### Descriptive and other statistical analyses

Frequencies, means and standard deviations (SD) were used to describe various sociodemographic, lifestyle, and anthropometric characteristics of cases and controls. Independent student t-test and Chi-square test were used to compare continuous and categorical variables respectively. Pearson’s correlation coefficients were used to examine the association between the derived dietary patterns and obesity markers (including BMI, waist circumference and percent body fat), energy, and energy adjusted nutrient intakes. Energy adjustment was carried out using the regression residual method [46]. The association between dietary patterns scores and odds of T2D was assessed using multivariate logistic regression models where the score of each dietary pattern was entered simultaneously as an independent variable in addition to other covariates. Covariates used in the logistic regression modeling included: energy, obesity markers (including BMI, waist circumference, percent body fat), age, sex, income, education, marital status, smoking, family history of T2D, physical activity, number of meals per day, and frequency of breakfast consumption. All analyses were two tailed and a *p*-value <0.05 was considered statistically significant. The Statistical Package for the Social Sciences was used for all computations (SPSS) (version 19.0, SPSS Inc., Chicago, Illinois, USA).

## Results

During the study period, 65 newly diagnosed cases of T2D were identified, and 58 agreed to participate in the study (response rate: 89%). Out of 139 control participants approached, 116 were enrolled in the study (response rate: 82%). The main reasons for declining participation were “lack of time” and “no interest”.

Table 1 presents the means and proportions of socio-demographic, lifestyle, and anthropometric characteristics of the study population. Mean age of the total study population was 56.18 ± 10.93 years (range: 40–79 years). Cases and controls were comparable across age, sex, income, marital status, education, and smoking. There were significant differences between cases and controls with regard to family history of T2D, physical activity, BMI, waist circumference, and percent body fat. Compared to controls, a higher proportion of cases reported a family history of T2D (58.6% vs. 13.8%). Cases who reported engaging in moderate to high physical activity levels were fewer than the controls (19.0% vs. 51.7%). Compared to controls, cases had a significantly higher mean BMI (30.79 ± 5.51 vs. 26.81 ± 4.48 Kg/m2), higher percent body fat (33.43 ± 8.76 vs. 28.88 ± 7.12), and a larger waist circumference (106.53 ± 12.01 vs. 95.92 ± 12.00 cm).

**Table 1:** Socio-demographic and anthropometric characteristics of the study population^a^

	**Total** (**n** = **174**)	**Cases** (**n** = **58**)	**Controls** (**n** = **116**)	**Significance**^**b**^
Age (years) (Mean ± SD)	56.18 ± 10.93	56.55 ± 11.20	55.99 ± 10.84	P-value > 0.05
Sex				X^2^ =0.00, P-value > 0.05
Males	105(60.3)	35(60.3)	70(60.3)	
Females	69(39.7)	23(39.7)	46(39.7)	
Income per month^c^				X^2^ = 1.521, P-value > 0.05
≤1.5 million LL	44(25.3)	18(31.0)	26(22.4)	
> 1.5 million LL	130(74.7)	40(69.0)	90(77.6)	
Marital status				X^2^ = 1.04, P-value > 0.05
Single	28(16.1)	7(12.1)	21(18.1)	
Married	146(83.9)	51(87.9)	95(81.9)	
Education				X^2^ = 3.85, P-value >0.05
< High school	31(17.8)	15(25.9)	16(13.8)	
≥ High school	143(82.2)	43(74.1)	100(86.2)	
Family history of diabetes				X^2^ = 37.94, P-value <0.001
None	124 (71.3%)	24 (41.4%)	100 (86.2%)	
Yes (mother, father or both)	50 (28.7%)	34 (58.6%)	16 (13.8%)	
Smoking				X^2^ = 0.12 , P-value > 0.05
Non smoker	120(69.0)	41(70.7)	79(68.1)	
Smoker ^d^	54(31.0)	17(29.3)	37(31.9)	
Physical activity level				X^2^ = 17.18 , P-value < 0.01
Low physical activity	103(59.2)	47 (81.0)	56(48.3)	
Moderate to high physical activity	71(40.8)	11(19.0)	60(51.7)	
Breakfast per week (Mean ± SD)	5.83 ± 2.12	5.72 ± 1.94	5.90 ± 2.22	P-value > 0.05
Meals per day (Mean ± SD)				X^2^ = 0.27, P-value > 0.05
1-2 meals per day	38(21.8)	14(24.1)	24(20.7)	
≥ 3 meals per day	136(78.2)	44(75.9)	92(79.3)	
Percent Body fat (%) (Mean ± SD)	30.40 ± 7.98	33.43 ± 8.76	28.88 ± 7.12	P-value < 0.001
BMI (Kg/m^2^) (Mean ± SD)	28.14 ± 5.19	30.79 ± 5.51	26.81 ± 4.48	P-value < 0.001
obese (BMI > =30)^e^	61(35.1)	32(55.2)	29(25.0)	X^2^ = 15.46, P-value < 0.001
Waist circumference (cm) (Mean ± SD)	99.45 ± 12.98	106.53 ± 12.01	95.92 ± 12.00	P-value < 0.001
Elevated waist circumference ^f^	145(83.3)	57(98.3)	88(75.9 )	X^2^ = 13.99, P-value < 0.001

^a^Categorical variables are expressed as n (%); continuous variables are expressed as Mean ± SD.

^b^Significance is derived from independent t-test for continuous variables and Chi square test for categorical variables.

^c^Income is expressed in terms of Lebanese Liras L.L (1500 L.L is almost equivalent to $1 U.S.).

^d^Smokers were defined as current smokers while non-smokers included non-smokers and past smokers.

^e^Obesity is defined as BMI ≥ 30Kg/m^2^.

^f^ Elevated waist circumference is defined by a circumference ≥ 94 cm for males and ≥ 80 cm for females according to the International Diabetes Foundation (IDF).

Factor analysis revealed four dietary patterns: Refined Grains & Desserts, Traditional Lebanese, Fast Food and Meat & Alcohol which explained 13.68%, 10.8%, 9.13%, and 8.67% of the dietary intake variance respectively (Table 2). The factor loading matrix derived from PCFA is shown in Table 2. The Refined Grains & Desserts pattern featured high consumption of pasta, desserts, fried fish, pizzas and pies, in addition to breakfast cereals and white bread. The second pattern, Traditional Lebanese, was mostly associated with consumption of olives and olive oil, fruits and vegetables, low fat milk and milk products, traditional Lebanese dishes, whole wheat bread, Arabic sweets, and “Shawarma” & ”Falafel”. The Fast Food pattern was characterized by high intakes of French fries, added fat, fast food sandwiches, mixed nuts, and full fat milk and milk products. The fourth pattern Meat & Alcohol loaded mostly on eggs, alcohol, red meat, juices and carbonated beverages, chicken, and fish (not fried). Eggs is often cited with meats as one food group (in most of the food guide pyramids, MyPlate, MyCedar, etc.), hence this pattern’s name “Meat & Alcohol” encompasses eggs as well.

**Table 2:** Factor loading matrix for the four identified dietary patterns in the study population^a, b^

**Food group**	**Patterns**			
	**Refined grains &desserts**	**Traditional Lebanese**	**Fast food**	**Meat &alcohol**
Pasta	**0**.**75**			
Desserts	**0**.**62**		0.25	
Fried fish	**0**.**58**			
Pizza and pies	**0**.**56**	0.21		
Breakfast cereals	**0**.**55**			
White bread	**0**.**53**		**0**.**25**	
Olives and olive oil		**0**.**67**		
Fruits		**0**.**60**	0.25	**0**.**35**
Vegetables		**0**.**59**		0.25
Low fat milk and milk products		**0**.**50**		
Traditional Lebanese dishes		**0**.**42**		
Whole wheat bread		**0**.**37**		
Arabic sweets	0.28	**0**.**37**		
Shawarma and Falafel		0.29		
French Fries			**0**.**60**	0.22
Added fat			**0**.**45**	
Fast food sandwiches			**0**.**57**	
Mixed nuts	0.21	0.29	**0**.**54**	0.24
Full fat milk and milk products	0.24		**0**.**41**	0.27
Eggs				**0**.**61**
Alcohol				**0**.**54**
Red meat				**0**.**54**
Sweetened juices and carbonated beverages			0.22	**0**.**33**
Chicken				**0**.**42**
Fish (not fried)				**0**.**23**
**Percent variance explained by each pattern**	**13**.**68**	**10**.**80**	**9**.**13**	**8**.**67**

^a^Absolute values < 0.2 were excluded from the table for simplicity.

^b^Absolute values ≥ 0.3 are bolded.

Table 3 shows the associations between the factor scores of the four dietary patterns with obesity markers, energy, and energy adjusted nutrient intakes as assessed by Pearson correlation coefficients. Nutrients which have been previously reported to be associated with T2D were included in this analysis. While the Refined Grains & Desserts, the Fast Food, and the Meat & Alcohol patterns were directly associated with BMI and waist circumference, inverse associations were noted between the Traditional Lebanese pattern and these obesity markers. Percent body fat was only directly associated with the Fast Food pattern (r = 0.249). The scores of the Refined Grains & Desserts, the Traditional Lebanese, the Meat & Alcohol patterns were directly correlated with energy, with those of the Refined Grains & Desserts pattern having the highest correlation (r = 0.741). In addition, the scores of the Refined Grains & Desserts pattern were directly correlated with total carbohydrates (r = 0.221) and sucrose (r = 0.161). The Traditional Lebanese pattern scores correlated directly with monounsaturated fat (r = 0.350) and dietary fiber (r = 0.411) intakes. The Fast Food pattern had the highest correlation with fat intake (r = 0.344). Compared to other patterns, the Meat & Alcohol pattern had the highest correlation with protein intake (r = 0.358).

**Table 3:** Pearson’s correlation coefficients between pattern scores and anthropometric measurements, total energy and energy adjusted nutrient intakes^a^

	**Refined grains &desserts**	**Traditional Lebanese**	**Fast food**	**Meat &alcohol**
BMI (Kg/m^2^)	0.173^*^	−0.236^**^	0.223^**^	0.159^*^
Waist circumference(cm)	0.233^*^	−0.262^**^	0.225^**^	0.237^**^
Percent body fat(%)	0.128	−0.024	0.249^**^	−0.070
Energy^b^(Kcal)	0.741**	0.095	0.428**	0.247**
Protein ( g)	−0.164*	−0.094	0.051	0.358**
Fat (g)	−0.099*	0.196*	0.344**	−0.135
Saturated fatty acids(g)	0.010	0.106	0.082	0.006
Mono unsaturated fatty acids(g)	−0.090	0.350**	0.232**	−0.060
Polyunsaturated fatty acids(g)	−0.124	0.009	0.203*	−0.051
α-Linolenic acid(g)	−0.048	−0.027	0.142	0.223**
Linoleic acid(g)	−0.120	−0.056	0.243**	−0.315**
Cholesterol(mg)	0.095	−0.129	−0.074	0.114
Carbohydrates(g)	0.221**	−0.050	0.009*	0.065
Dietary fibers(g)	0.143	0.411**	0.002	−0.148
Sucrose(g)	0.161*	0.099	0.005	0.083

^a^Adjustment for energy was done by residual method described by Willet [46].

^b^Absolute values are indicated for the correlation of dietary pattern scores with total energy intake.

* Correlation is significant at p < 0.05.

**Correlation is significant at p < 0.01.

Table 4 showed the association between the identified dietary patterns scores with the odds of T2D. In the final multivariate logistic regression model (adjusted for energy, sociodemographic and lifestyle characteristics, BMI, waist circumference, and percent body fat), a unit increase in the scores of the Refined Grains & Desserts pattern was associated with more than 3 folds increase in the odds of T2D (OR: 3.85, CI: 1.31-11.23). Similarly, scores of the Fast Food pattern were associated with higher odds of T2D (OR: 2.80, CI: 1.41-5.59). The odds of T2D were lower with increasing scores of the Traditional Lebanese pattern (OR: 0.46, CI: 0.22-0.97). No significant association was noted between the scores of the Meat & Alcohol dietary pattern and the odds of T2D.

**Table 4:** Odds ratios and 95% confidence intervals for the association between dietary patterns and the odds of T2D in the study population

	**Refined grains &desserts**		**Traditional Lebanese**		**Fast food**		**Meat &alcohol**	
	**OR**	**95%CI**	**OR**	**95%CI**	**OR**	**95%CI**	**OR**	**95%CI**
Model 1^*^	2.94	1.26–6.84	0.53	0.31–0.91	2.09	1.28–3.41	1.05	0.69–1.59
Model 2^**^	3.22	1.17–8.84	0.60	0.33–1.08	2.32	1.32–4.06	1.19	0.71–1.98
Model 3^***^	3.85	1.31–11.23	0.46	0.22–0.97	2.80	1.41–5.59	1.43	0.83–2.46

^*^Model 1 is adjusted for age (in years), sex (male, female), waist circumference (in cm), body fat (percentage), BMI (in Kg/m^2^), and energy (Calories).

^**^Model 2 is adjusted for variables in model 1 as well as income per month (≤1.5 million L.L., >1.5 million L.L.; 1500 L.L is almost equivalent to $1 U.S.), smoking (smokers including current smokers, non smokers including non-smokers and past smokers), family history of T2D (yes vs. no), education (less than high school education and greater than high school education), marital status (single including single, divorced, and widowed, and married including currently married or living with a partner).

^***^Model 3 is adjusted for variables in model 1 and model 2 as well as physical activity (low vs. middle to high levels), meals per day (1–2 vs. ≥ 3 meals per day), breakfast (frequency per week).

## Discussion

This case control study aimed at evaluating the association between dietary patterns and the odds of T2D in a sample of 58 recently diagnosed cases with the disease and 116 matched controls residing in Beirut, the capital of Lebanon. Studying the association between dietary patterns and T2D overcomes the inconsistent findings of single nutrient analysis in evaluating diet-disease associations, takes into consideration the synergistic effects of nutrients, and produces culture specific recommendations. Furthermore, behavior change could be better achieved when discussed in terms of patterns, rather than nutrients or specific foods, because it gives food context, which in turn may facilitate the development of public health recommendations [16]. Our results revealed four major dietary patterns in this study population: Refined Grains & Desserts, Traditional Lebanese, Fast Food and Meat & Alcohol patterns which together explained 42.28% of the total variance in the dietary intake. The percentage of variance explained by the first four dietary patterns identified is similar in magnitude to what has been reported in others studies [47,48]. It should be noted, however, the variance explained by all factors is a function of the number of factors researchers choose to retain [49]. Both the Refined Grains & Desserts and the Fast Food patterns were associated with higher odds of T2D, while the Traditional Lebanese was inversely associated with the odds of the diseases. No association was observed between the Meat & Alcohol pattern with the odds of T2D.

Previous studies have reported on the association between dietary patterns and the risk of T2D [21-24,50]. Overall, their results featured two main dietary patterns, the Western and Prudent. The Western pattern, characterized by a high consumption of red meat, eggs, refined grains, desserts, French fries, and high fat dairy products, was associated with higher plasma insulin [47,51], C-peptide levels [52], and overall risk of T2D [19,21,47,51]. A recent cohort study showed that adherence to a Western pattern characterized by high intakes of desserts, meats, and refined grains during adolescence was associated with 29% increase in the risk of T2D in middle aged women [53]. The Prudent pattern contained high amounts of fruits, vegetables, legumes, fish, poultry, and low fat dairy products and was not always reported as associated with reduced risk of disease [19,21,23,25]. Hence, the association between the prudent pattern and the risk of T2D was less evident than that of the Western pattern with the risk of disease. Data describing the association between dietary patterns and T2D in the MENA region are scarce and limited to countries with particular patterns that are country and culture specific [33,34]. In the present study, the Refined Grains & Desserts and the Fast Food patterns were the two patterns shown to have a direct association with the odds of T2D. Both of these patterns shared important features with the Western pattern previously described in the literature and shown to directly influence the risk of T2D [17,21-27]. The Refined Grains & Desserts pattern, found in this study, was characterized by high intake of pasta, desserts, pies, and pizzas, in addition to breakfast cereals and white bread and was directly correlated with energy and carbohydrates intake. The Fast Food pattern was characterized by high intake of chips and fries, fast food sandwiches, full fat milk and milk products, and added fats, and was correlated mainly with energy and fat intake. There are a few mechanisms by which adherence to these patterns may increase the risk of T2D. Many of the foods and food groups loading high on these patterns possess a high glycemic index as well as high glycemic load. Such foods have been repeatedly reported to have a direct association with T2D [54-61]. Foods with a high glycemic load trigger higher postprandial insulin levels, which may lead to an accelerated age related decline in insulin secretion and ultimately the onset of T2D [13]. Furthermore, high consumption of sugar has been independently associated with the odds of T2D [62,63]. In line with the findings of this study were the results of the National Nutrition Survey conducted in Lebanon in 2009, which indicated an association between a Western dietary pattern and increased odds of obesity [41], and the metabolic syndrome in Lebanon, conditions postulated to increase the risk of T2D [28].

The Traditional Lebanese pattern identified in this study was inversely associated with the odds of T2D. Although Mediterranean diets could be different from one country to another in the Mediterranean basin [64], the Traditional Lebanese pattern encompasses many features of the generally known Mediterranean diet. These features include consumption of fruits, vegetables, olives and olive oil, and whole bread [65]. Adherence to a Mediterranean diet has been repeatedly shown to exert a beneficial role regarding the development and progression of T2D [29-32,66]. Additionally, individual food groups and components of the diet, such as monounsaturated fatty acids, fruits, vegetables, and whole grain cereals may protect against the development of diabetes, possibly through the amelioration of insulin sensitivity and their anti-inflammatory actions [67]. A recent study among adults in rural Lebanon indicated that adherence to a Mediterranean diet, as assessed using the widely used Mediterranean diet score (MDS), is negatively associated with two of T2D known risk factors: obesity and visceral adiposity [68].

Our results indicated that the Meat & Alcohol pattern did not show an association with the odds of T2D. This pattern included food groups that were shown to be associated with varying odds of T2D. For instance, frequent consumption of meat, in particular red meat, has been reported to increase the risk of diabetes in prospective studies [21,69,70]. Furthermore, recent evidence, though inconclusive, suggested that increased consumption of eggs may raise blood cholesterol concentrations and thereby increase the risk of T2D by impairing islet cell function [71-73]. On the other hand consumption of fish was shown to decrease the risk of the disease [50]. N-3 fatty acids (including α-Linolenic acid found to be positively correlated with the scores of this pattern) have been suggested to have beneficial effects on health outcomes such as reduction in concentrations of inflammatory markers and improvement of insulin sensitivity [74]. This inverse association between fish intake and the risk of T2D was challenged by a recent meta-analysis by Patel et al., which showed that lean fish, total fish, and shellfish intakes were not associated with incident diabetes but that fatty fish intake may be weakly inversely associated [75]. Moderate alcohol consumption (1–3 drinks per day) has been shown to be associated with lower risk of diabetes [76] and lower levels of pro inflammatory markers [77]. Hence the negative effects of meat and egg consumption on the odds of T2D could have been neutralized by the protective effect fish and moderate alcohol consumption might have had on the odds of the disease.

A few limitations should be considered when interpreting the findings of this study. The recall of past diet rises concerns regarding the possibility of recall bias among cases if their diet changed as a result of their diagnosis, or because they experienced symptoms as a result of the presence of subclinical disease before diagnosis. It is also possible that the dietary intake interview reflected changes due to medical advice. To minimize this recall bias, cases were constantly reminded to report on their dietary intake before diagnosis with T2D. Another limitation stems from the use of factor analysis in the derivation of the dietary patterns. This method involves many subjective decisions in selecting and grouping foods for analysis from the questionnaire, in determining the number of factors to retain, in choosing the method of rotation of the initial factors and in labeling dietary patterns according to their factor loadings [78]. To address the issue of subjectivity using factor analysis, the food groupings used in this study were similar to those reported by others in the region [28,33,34,41] and the selection of the factor solution was done after evaluating the scree plots and eigenvalues. It remains important to note that the food frequency questionnaire used in this study was not validated in our study population. The fact that the food frequency questionnaire was administered by a trained dietitian and not self-completed offers many advantages in that it does not require a literate population and results in consistent interpretations and higher response and completion rates, each of which may enhance the validity of the data [78,79]. Furthermore, any non-differential measurement error of the diet is likely to have attenuated the associations that we have observed. Finally, although significant associations were found between the Refined Grains & Desserts, the Traditional Lebanese and the Fast Food patterns with the odds of T2D, the small sample size of this study might have lead to insufficient power to detect an association between the Meat & Alcohol pattern and the disease.

## Conclusion

This study provides insight to the association between dietary patterns and T2D, particularly among Lebanese adults, a currently understudied population. Our findings suggested that Refined Grains & Desserts and Fast Food dietary patterns were associated with increased odds of T2D while a Traditional Lebanese pattern was indirectly associated with the odds of the disease. Thus our results allow for evidence-based recommendations to limit the consumption of refined grains products, desserts, fast food sandwiches, French fries, and added fats as well as encouraging the consumption of a Traditional pattern based on fruits and vegetables. These recommendations lay grounds for planning intervention strategies that are culture based and in line with local dietary habits. The findings of this study are important not only in Lebanon but also in other countries of the Middle East region where such patterns are predominantly available.

## Abbreviations

T2D: Type 2 Diabetes OR, Odds Ratio; CI: Confidence Interval; MENA: Middle East North Africa; FFQ: Food Frequency Questionnaire; BMI: Body Mass Index; BIA: Leg to leg bioelectric impedance; N4: N-squared Computing Nutritionist IV; PCFA: Principal component factor analysis; SD: Standard Deviation; SPSS: Statistical Package for the Social Sciences; r: Correlation coefficient.

## Competing interests

The authors declare that they have no competing interests.

## Authors’ contributions

FN, as the principal investigator, was responsible for the concept and design of the study. NH supervised data collection and analysis. LI was responsible for the statistical evaluation of data. MS undertook the dietary assessment of study participants. SA participated in the statistical analysis and data interpretation. MNZ participated in the design of the study and supervision. LN played a central role in integrating the dietary and anthropometric results and acted as lead reviewer. All authors participated in the preparation of, and have approved the final version of the manuscript.

## Supplementary Material

Additional file 1 **Appendix A.** Food groups used in the analysis of the dietary patterns. Click here for file
