# Supplementary material for: Dietary patterns and odds of Type 2 diabetes in Beirut, Lebanon: a case–control study
Source: Nutr Metab (Lond). 2012 Dec 27;9:111. doi: 10.1186/1743-7075-9-111 (PMC3565896; doi:10.1186/1743-7075-9-111)
Supplement: Additional file 1 — Appendix A. Food groups used in the analysis of the dietary patterns. [file 1743-7075-9-111-S1.doc]

Appendix A: Food groups used in the analysis of the dietary patterns

| Added fat | Butter, “Tehineh”, vegetable oil, mayonnaise |
| --- | --- |
| Alcohol | Beer, none wine, wine. |
| Arabic sweets | “Baklava” and derivatives including traditional puff pastry with “Kashta” and melted cheese. |
| Breakfast cereals | All types of breakfast cereals. |
| Chicken | chicken (Fried, grilled, broiled, stir fried) |
| Desserts | Honey, added sugar, all kinds of jam, molasses, fruit pies, croissants, doughnuts, ice cream, chocolate, pudding, biscuits and cakes |
| Eggs | All types of eggs |
| “Shawarma” and “Falafel” sandwiches | “Falafel” and “Shawarma” eaten as a side dish or in a sandwich. Shawarma refers to a meat preparation where lamb, goat, chicken, turkey, beef, or mixed meats are grilled on a rotating vertical skewer. Meat shavings of this skewer are then served. |
| Fast food sandwiches | Hamburgers, Fahitas, Philadelphia, chicken and meat escalope sandwiches |
| Fried Fish | Fried fish |
| Fish | Fish (grilled, broiled, stir fried) |
| French fries | French fries, potato chips regular, potato chips light |
| Fruits | Dried fruits, fresh fruits, fresh fruit juices, and fruit juices made from concentrate |
| Full fat milk and milk products | Full fat cheese , processed cheese, whole milk, whole fat ”Labneh”, whole fat yogurt |
| Sweetened juices and carbonated beverages | Sweetened fruit juice, soda, diet soda |
| Low fat milk and milk products | Low fat cheese, zero fat milk, low fat milk, “Labneh” light, yogurt light, zero fat “Labneh” |
| Mixed Nuts | All kinds of nuts (raw or roasted) |
| Olives and olive oil | Olives and olive oil (used fresh, i.e. not used for frying) |
| Pasta | All kinds of pasta |
| Pies and pizzas | All types of pizzas, pies and “Manaeesh” (Lebanese version of pizza) |
| Red meat | All types of red meat such as lamb and beef (Fried, grilled, broiled, stir fried) |
| Traditional Lebanese mixed dishes | Traditional Lebanese mixed dishes include two types of dishes: vegetable stew with rice (artichoke, eggplant, cauliflower, chicory, fried with onions, green beans , jew’s mallow, peas, okra spinach) or stuffed vegetables including: eggplants, grape leaves, zucchini and cabbages leaves in addition to “Burghol” (crushed wheat) |
| Vegetables | Raw vegetables |
| White bread | White bread, kaak (dried bread), toast and crackers |
| Whole wheat bread | Whole wheat bread, brown toast and whole wheat crackers |
